# Cortical bone adaptation to a moderate level of mechanical loading in male *Sost* deficient mice

**DOI:** 10.1038/s41598-020-79098-0

**Published:** 2020-12-18

**Authors:** Haisheng Yang, Alexander Büttner, Laia Albiol, Catherine Julien, Tobias Thiele, Christine Figge, Ina Kramer, Michaela Kneissel, Georg N. Duda, Sara Checa, Bettina M. Willie

**Affiliations:** 1 Department of Biomedical Engineering, Faculty of Environment and Life, Beijing University of Technology, Beijing, China; 2 Julius Wolff Institute and BIH Center for Regenerative Therapies, Berlin Institute of Health and Charité-Universitätsmedizin Berlin, Berlin, Germany; 3 Research Centre, Shriners Hospital for Children-Canada, 1003 Decarie Blvd, Montreal, QC H4A 0A9 Canada; 4 Novartis Institutes for BioMedical Research, Basel, Switzerland; 5 Department of Pediatric Surgery, McGill University, Montreal, Canada

**Keywords:** Osteoporosis, Preclinical research

## Abstract

Loss-of-function mutations in the *Sost* gene lead to high bone mass phenotypes. Pharmacological inhibition of *Sost*/sclerostin provides a new drug strategy for treating osteoporosis. Questions remain as to how physical activity may affect bone mass under sclerostin inhibition and if that effect differs between males and females. We previously observed in female *Sost* knockout (KO) mice an enhanced cortical bone formation response to a moderate level of applied loading (900 με at the tibial midshaft). The purpose of the present study was to examine cortical bone adaptation to the same strain level applied to male *Sost* KO mice. Strain-matched in vivo compressive loading was applied to the tibiae of 10-, 26- and 52-week-old male *Sost* KO and littermate control (LC) mice. The effect of tibial loading on bone (re)modeling was measured by microCT, 3D time-lapse in vivo morphometry, 2D histomorphometry and gene expression analyses. As expected, *Sost* deficiency led to high cortical bone mass in 10- and 26-week-old male mice as a result of increased bone formation. However, the enhanced bone formation associated with *Sost* deficiency did not appear to diminish with skeletal maturation. An increase in bone resorption was observed with skeletal maturation in male LC and *Sost* KO mice. Two weeks of in vivo loading (900 με at the tibial midshaft) induced only a mild anabolic response in 10- and 26-week-old male mice, independent of *Sost* deficiency. A decrease in the Wnt inhibitor *Dkk1* expression was observed 3 h after loading in 52-week-old *Sost* KO and LC mice, and an increase in *Lef1* expression was observed 8 h after loading in 10-week-old *Sost* KO mice. The current results suggest that long-term inhibition of sclerostin in male mice does not influence the adaptive response of cortical bone to moderate levels of loading. In contrast with our previous strain-matched study in females showing enhanced bone responses with *Sost* ablation, these results in males indicate that the influence of *Sost* deficiency on the cortical bone formation response to a moderate level of loading differs between males and females. Clinical studies examining antibodies to inhibit sclerostin may need to consider that the efficacy of additional physical activity regimens may be sex dependent.

## Introduction

Loss-of-function mutations in the *Sost* gene lead to osteosclerotic phenotypes in humans (sclerosteosis, OMIM 269500 and van Buchem disease, OMIM 239100)^1,2^ and to high bone mass phenotypes in male and female mice^3,4^. Interestingly, female *Sost* null mice have an increased volumetric cortical bone mineral density compared to males^3^. In contrast, transgenic female mice overexpressing *Sost* exhibit low bone mass^5^. *Sost* is a negative regulator of Wnt signaling and subsequently bone formation, since canonical Wnt signaling activation increases bone mass. Pharmacological inhibition of sclerostin, the product of the *Sost* gene, through a sclerostin-neutralizing antibody (Scl-Ab) has shown significant bone gains in rodents^6^ and nonhuman primates^7^ and reduced fracture risk in postmenopausal women with osteoporosis^8^. Romosozumab (Evenity™, Amgen and UCB) was recently approved by the FDA for the treatment of postmenopausal women with osteoporosis at high risk of fracture. Other sclerostin antibodies are being tested for use in children and adults with rare diseases, such as Type I, III or IV Osteogenesis Imperfecta (Setrusumab, formerly called BPS804, Mereo BioPharma).

Questions remain as to how physical activity may enhance bone mass in young and old male and female patients undergoing sclerostin inhibition treatment. Fracture risk is of course a concern in the clinical populations where sclerostin antibodies will be used, thus moderate rather than high intensity training regimes are likely more feasible. Nevertheless, it is important to understand how the adaptive response to in vivo loading is altered by sclerostin inhibition across age and sex. Sclerostin is primarily expressed by the osteocytes and mechanical loading of cortical bone in the mouse ulnae led to substantial downregulation in endogenous *Sost* mRNA expression and a decreased number of stained osteocytes releasing sclerostin^9^. It has been suggested that loading-induced down-regulation of sclerostin increases bone formation by relieving inhibition of canonical Wnt signaling in osteoblasts as well as by suppressing resorption through the regulation of OPG. We recently showed female *Sost* knockout (KO) mice had a significantly greater load-induced cortical (mid-diaphysis) and trabecular (metaphysis) bone formation response compared to female littermate control (LC) mice^10,11^. *Dkk1* expression was significantly greater in female *Sost* KO compared to LC mice, and was significantly downregulated with loading in both female *Sost* KO and LC mice. The load-induced cortical resorption response was similar in female *Sost* KO and LC mice. Others have reported region-specific load-induced cortical bone formation response in the absence of *Sost* in young female mice^12,13^. It was shown that sclerostin antibody treatment enhances the anabolic bone formation response to loading in young female C57BL6 mice^14^.

We have previously shown a net gain in trabecular bone volume fracture and trabecular thickness could be achieved with loading in *Sost* KO adult female mice, due to reduced resorption, which was not the case in males^11^. It is unknown how Wnt signaling is affected during loading under *Sost* inhibition in male mice, since all of the aforementioned studies were performed in females and our single study in males did not examine gene-level changes in the trabecular bone. This is important since previous studies have shown that in addition to aging, sex has an influence on Wnt/β-catenin signaling in bone, suggesting that sex hormones may be possible regulators or mediators of sclerostin function^15^. Modder et al. reported that men have higher serum sclerostin levels than women and that serum sclerostin levels increase markedly with age^16^. Callewaert et al. showed that disrupting androgen receptor signaling increased periosteal bone formation following ulnar loading, in addition to lowering *Sost* and sclerostin expression after loading (more than in wildtypes)^17^. They suggested that androgen receptor signaling may interfere with the inhibiting effect of sclerostin signaling on bone formation^18^. Several studies have revealed a sex-related difference in bone mass and/or the anabolic response to mechanical loading in mice that had altered Wnt/β-catenin signaling. Deletion of a single copy of β‐catenin in osteocytes abolished the anabolic response to loading in male and female mice, but interestingly the osteocyte β-catenin haploinsufficiency led to reduced bone mass in 8- and 20-week-old females, with less effect on males^19,20^. Sex differences were observed in the anabolic response to loading of *Lrp5* null and *Lrp5HBM* + mice^21,22^. Female Lrp5-deficient mice had greater suppression of exercise-dependent bone remodeling than males^21^, while female *Lrp5HBM* + mice were more responsive to lower magnitudes of loads and had less bone loss in response to disuse compared to males^23^. These results demonstrate the need for further examination of the underlying basis for the observed sexual dimorphism in light of sclerostin inhibition that targets this pathway.

Since the majority of pre-clinical loading studies to date focused on females, it remains unclear if physical activity will be beneficial in older as well as young males undergoing sclerostin inhibition. Less is known in general regarding how males with or without *Sost* deficiency respond to mechanical loading with age. Ducher et al. showed that periosteal bone formation in male tennis players’ humeri was more pronounced in prepubertal and peripubertal boys and plateaued in postpubertal players^24^. Mosley et al. reported no sex-related differences in the cortical bone formation response to ulnar loading in young rats^25^, while other studies observed an elevated mechanoresponse in cortical bone of female wild-type mice^26–29^. We observed that the cortical bone formation response to loading was reduced at maturation in both female LC and *Sost* KO mice coincident with age-dependent expression of Wnt target genes in female *Sost* KO and LC mice as well as *Sost* gene expression in LC mice^10^. It remains unclear if male *Sost* KO and LC mice have age-related changes in mechanoresponse similar to what has been reported in female wild-type mice^30–33^. This knowledge is especially relevant considering that men suffer from age-related bone loss and have fragility fractures and thus would benefit from sclerostin inhibition^34^.

Motivated by our earlier observations in females^10^, we hypothesized that (1) *Sost* deficiency in male mice would lead to an enhanced anabolic response to loading and (2) the bone formation response in males would be reduced at skeletal maturation. To test these hypotheses, we first performed in vivo strain gauging on male young growing (10-week-old), adult skeletally mature (26-week-old) and middle-aged (52-week-old) mice, and developed finite element (FE) models for the young and adult mice. We then applied two weeks of in vivo dynamic axial compressive loading to the tibiae of male young and adult *Sost* KO and LC mice, and quantified changes in cortical bone morphology by microCT, bone formation and resorption by 3D time-lapse in vivo morphometry using registered microCT data and conventional 2D histomorphometry. We also measured the gene expression of several Wnt target genes and Wnt inhibitors at 3, 8, and 24 h after a single loading session in male young and middle-aged mice of both genotypes. It should be noted that we did not examine gene expression in adult mice, but focused only on young and middle-aged mice to see a potential greater difference. Also, we did not examine the cortical bone formation/resorption changes in response to loading over two weeks in middle-aged male mice, since the load levels required to engender known osteogenic strain levels (e.g. 900 µɛ) were discovered to be above those that led to limping and joint damage in previous pilot studies in female *Sost* KO mice^10^.

## Materials and methods

### Animals

Sperm from four male *Sost* -/- mice was provided by Novartis. Intracytoplasmic sperm injection with the oocytes from female C57BL/6 J mice was performed and a breeding colony was maintained. The first heterozygous generation was mated among themselves. In following generations, homozygous *Sost* KO and LC mice were identified using a Multiplex PCR with mice tail cuts, according to a protocol provided by Novartis. *Sost* KO and LC male mice at age of 10, 26 and 52 weeks were used in this study. All animal experimental procedures were approved by the local animal welfare ethics committee (LAGeSo Berlin, G0021/11). All methods were carried out in accordance with relevant guidelines and regulations of the local animal welfare ethics committee.

### In vivo* load-strain calibration*

To conduct a strain-matched in vivo tibial loading experiment, age- and genotype-specific load-strain relationships were determined by a separate strain gauging experiment (n = 7/age/genotype; 42 mice in total). At 10, 26, and 52 weeks of age, single element strain gauges (EA-06-015LA-120/E2, Micromeasurements, USA) were surgically attached to the medial surface of the tibial midshaft in LC and *Sost* KO male mice while mice were under anesthesia. A range of axial dynamic compressive loads (peak loads ranging from − 2 to − 14 N in LC and − 2 to − 20 N in *Sost* KO mice) was applied to the tibia using a custom-made fixture built in a loading device (Testbench Electro Force LM1, TA Instruments, USA), while strain measurements were recorded simultaneously using WinTest software associated with the loading device. Strain gauge measures were performed on both left and right tibiae of each mouse. In vivo stiffness (load/strain) was calculated as the change in load over the change in strain as described previously^35,36^ and used to calculate the peak compressive load required to induce + 900 με at the gauge site of the tibial midshaft (Supplementary Table 1).

### Finite element analysis

Since the strain gauging experiment only provided strain measures at a very local area (gauge site) on bone surface and the gauge-measured strain does not always represent the peak or average strain at the tibial midshaft, microCT-based FE analyses were conducted to determine the load-induced strain environment across the entire tibia. The FE modeling approach used here had been developed and validated in our previous studies examining female C57BL/6 and female *Sost* KO mice^37–39^. Briefly, FE models of typical 10- and 26-week-old *Sost* KO and LC tibiae (n = 1/age/genotype) were built based on ex vivo microCT scanning of the strain-gauged whole tibiae (Amira, Thermo Fisher Scientific), at an isotropic voxel resolution of 9.91 μm (Skyscan 1172, Brukers, Belgium; 100 kVp, 100 µA, 360°, 0.3° rotation step, 3 frame averaging). Heterogeneous material properties (Young´s modulus) based on spatial distribution of tissue mineral density in the microCT scans were assigned to the FE models consisting of tetrahedral elements^38–40^. Poisson´s ratio was set to 0.35 for all models^39^. Loading and boundary conditions were applied to mimic the experimental in vivo axial loading of the tibia. Linear elastic FE analyses were performed (Abaqus 6.13, Dassault Systemés Simulia, MA). The predicted strain value at the gauge site for each tibia was calculated by averaging the strain in the longitudinal direction of the strain gauge at its mounting position, which was visible on the scans. The predicted strains in cortical bone were calculated in a volume of interest (VOI) centered at the tibia midshaft, containing 5% of the total tibia length, analogue to the region used for the microCT analysis. The maximum absolute value between the maximal (tensile, ε_Max_) and minimal (compressive, ε_Min_) principal strains was calculated for each element within the heterogeneous FE models. Thereafter, the mean values of the tensile ($\bar{\varepsilon }_{\text{Max}}$) and compressive ($\bar{\varepsilon }_{\text{Min}}$) strains in the midshaft VOI were determined. Elements for which abs (ε_Max_) > abs (ε_Min_ ) were used to calculate $\bar{\varepsilon }_{\text{Max}}$ and SD (ε_Max_), while elements for which abs (ε_Max_) ≤ abs (ε_Min_ ) were used to calculate $\bar{\varepsilon }_{\text{Min}}$ and SD (ε_Min_). The SD (standard deviation) reflects the range of ε_Max_ for tension and ε_Min_ for compression within the VOI.

### In vivo tibial mechanical loading

**Figure 1:**
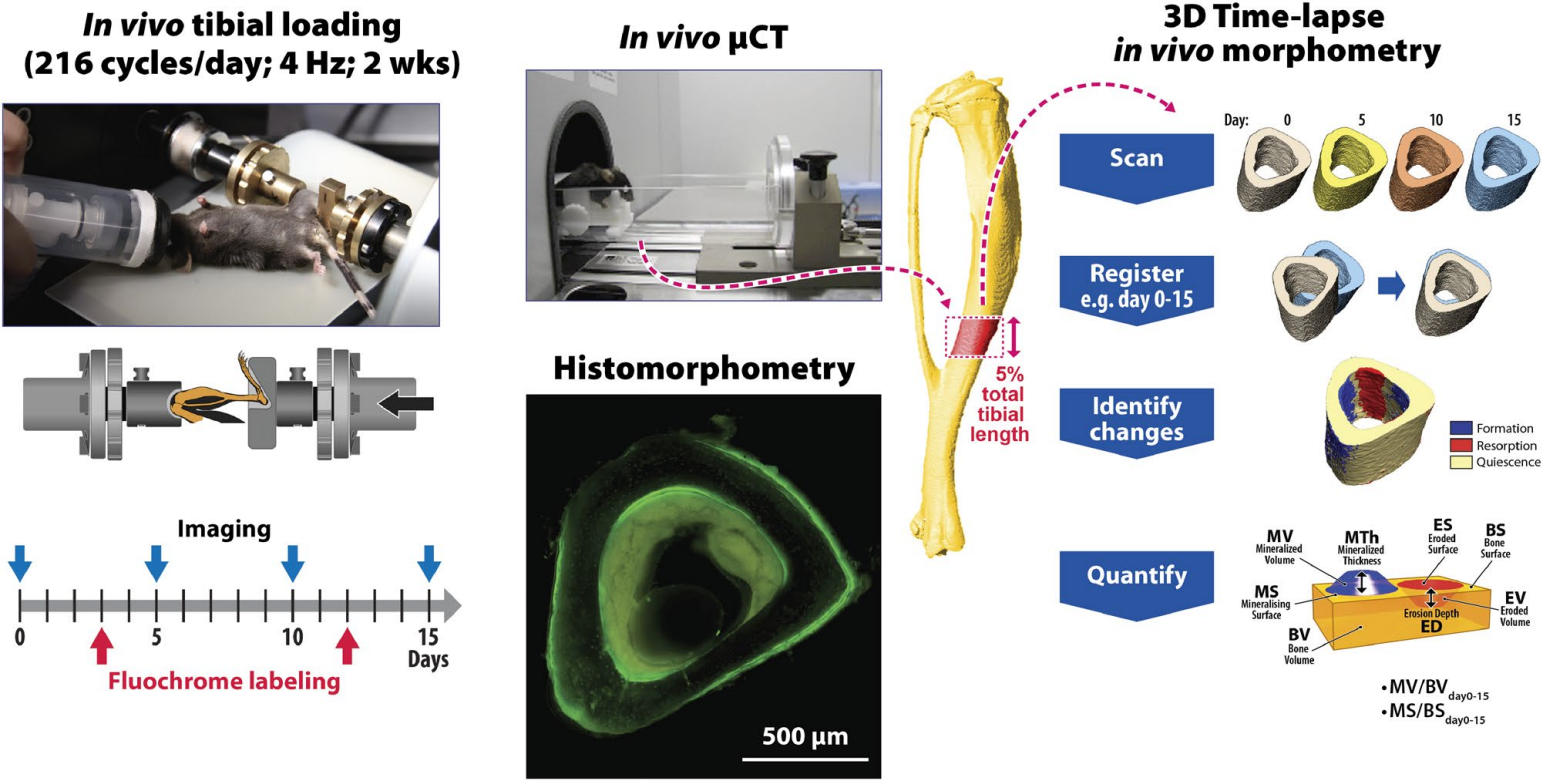
Schematic illustration of the experimental in vivo loading setup, including timeline for in vivo microCT imaging (day 0, 5, 10, 15), fluorochrome labeling for histomorphometry and procedure for 3D time-lapse in vivo morphometry. The region of interest for microCT analysis, 3D time-lapse in vivo morphometry and conventional histomorphometry was centered at the tibial midshaft.

The left tibiae of 10- and 26-week-old *Sost* KO and LC mice (n = 8/age/genotype) underwent daily in vivo cyclic compressive loading (900 με at strain gauge site, 216 cycles/day at 4 Hz) for 2 weeks (5 days/week, Monday-Friday) (Fig. 1). The strain level of 900 με was the same as that used in our previous study examining the effect of *Sost* deficiency on cortical and trabecular bone adaptation in female mice^10,11^. Although this strain is lower than the more commonly used peak strain of 1200 με, it is higher than strain levels engendered in wild-type C57BL6 mice during normal walking (200–600 µɛ)^41,42^. The loading frequency (4 Hz) represents the stride frequency of the mouse^43^. The loading regime included triangle waveforms with 0.15 s symmetric active loading/unloading, a 0.1 s rest phase (at − 1 N) between every load cycle and a 5 s rest inserted between every four cycles^35^. At day 15, the mice were euthanized through an overdose of potassium chloride while under anesthesia (ketamine 60 mg/kg and medetomidine 0.3 mg/kg). The weight was measured daily throughout the experiment and no abnormal weight loss was observed; there was no significant difference in start or end weight within a group (Supplementary Table 2). No mice exhibited any complications or signs of limping throughout the loading experiment. After dissection, tibial bone length was measured (Supplementary Table 2).

### Longitudinal in vivo microCT

In vivo microCT with a voxel size of 10.5 μm was performed on anesthetized mice at day 0, 5, 10, and 15 to assess the midshaft cortical bone of both the right and left tibiae (vivaCT 40, Scanco Medical, Switzerland; 55 kVp source voltage, 145 μA source current, 300 ms integration time, no frame averaging, range of 180 degrees) (Fig. 1). To prevent motion artifacts during microCT scanning, anaesthetized mice were constrained in a custom-made plastic mouse bed. Our previous study has shown the repeated radiation exposure (0, 5, 10, 15 day) from microCT at doses used in this study (approximately 0.48 Gy per scan) does not affect cortical bone parameters measured by microCT or histomorphometry in 10 and 26 week-old C57BL/6 mice^35^.

### In vivo microCT analysis

Similar to previous studies, midshaft cortical bone (5% of tibial length) was analyzed from microCT images at day 0, 5, 10 and 15 using the IPL (Image Processing Language) based standard evaluation scripts provided by Scanco (Scanco Medical, Switzerland) (Fig. 1). Although whole-tibia analyses from previous tibial loading studies have observed relatively robust mechanoadaptive responses along ~ 10–60% of the tibia from its proximal end^44–46^, the midshaft was chosen here because it is a common location where strain gauging is performed and load-strain relations accounting for the effect of age, gender, genotype, etc. are established. It is also a common volume of interest for examining cortical bone adaptation in the tibial loading model^32,33,35,41,46–48^. A global threshold of 4626 HU (809.6 mg HA/ccm) was used to segment cortical bone from soft tissue and water in all mice. The measured cortical bone parameters included: principal moments of inertia (I_max_, I_min_), cortical bone area (Ct.Ar), total cross-sectional area (T.Ar), cortical area fraction (Ct.Ar/T.Ar), cortical thickness (Ct.Th), and cortical tissue mineral density (Ct.TMD).

### Time-lapse in vivo morphometry

MicroCT images taken at day 0 and 15 were geometrically aligned and analyzed using a registration, segmentation and quantification algorithm using Amira software (Thermo Fisher Scientific) (Fig. 1). The method has previously been described in detail^30,49,50^. Briefly, the algorithm involves the following steps: (1) geometrical registration of images, (2) thresholding to extract the bone region, using the same global threshold mentioned above, (3) segmentation to exclude mineralized tissue present in the medullary cavity inside the VOI, (4) labeling regions of quiescent, newly formed and resorbed bone, and (5) quantification of volumetric dynamic (re)modeling parameters of formation and resorption normalized to values at the beginning of experiment (bone volume newly mineralized between day 0 and 15 divided by the bone volume present at day 0: MV/BV_day0–15_; bone volume eroded between day 0 and 15 divided by the bone volume present at day 0: EV/BV_day0–15_; mineralizing and eroded surface between day 0 and 15 normalized to the total bone surface at day 0: MS/BS_day0–15_ and ES/BS_day0–15_). The dynamic bone formation and resorption parameters were also quantified for the endocortical and periosteal surfaces, separately using previously described methods^51^.

### Histomorphometry

Calcein (20 mg/kg) was administered to the LC and *Sost* KO mice via intraperitoneal injection at day 3 and 12 during the loading experiment (Fig. 1)^52^. The tibiae were dehydrated in ascending grades of ethanol to absolute, cleared in xylene, infiltrated and finally embedded in polymethyl-methacrylate. The blocks were sectioned transversal to the bone’s long axis at the cortical midshaft. The slices were ground and polished to an approximately thickness of 60 μm and viewed at a magnification of 200 × under a mercury lamp microscope (KS400 3.0, Zeiss, Germany) for evidence of fluorochrome labels (Fig. 1). Images were acquired using commercially available software (Axiovision, Zeiss, Germany). The analyzed region of interest for the cortical bone included endocortical and periosteal surface. The single- and double-labeled surface per bone surface (sLS/BS, dLS/BS), mineralizing surface (MS/BS), mineral apposition rate (MAR), and bone formation rate (BFR/BS), were analyzed as recommended and using ImageJ^3^. MS/BS was calculated as 0.5 × sLS/BS + dLS/BS. When a specimen had no double-labeled surface (dLS/BS = 0), it was labeled as “no data” for MAR and BFR/BS^53^. The amount of newly mineralized bone per day was calculated using the averaged double label distances divided by the 9-day labeling interval and expressed as the MAR in units of microns per day. For determining MAR, the entire endocortical (Ec) and periosteal (Ps) surfaces were analyzed.

### qPCR analysis

The left tibiae of additional groups of 10-week-old and 52-week-old mice (n = 6/genotype/age/time point following loading) were loaded for a single loading session. The loading procedure has been described above (see section In vivo tibial mechanical loading). Mice were sacrificed at either 3, 8, or 24 h after the single loading session. Left loaded and right nonloaded tibiae were analyzed for gene expression. The ends of the bone and the bone marrow was removed and RNA was extracted using the Aurum total RNA fatty and fibrous tissue kit (Bio-Rad). RNA quality and concentration were verified by NanoDrop and reverse transcription was performed using the High Capacity cDNA Reverse Transcription Kit (Thermo Fisher Scientific). Gene expression was determined using a QuantStudio 7 Flex real-time PCR system and the following TaqMan probes (Thermo Fisher Scientific): *Lef1* (Mm00550265_m1), *Axin2* (Mm00443610_m1), *Dkk1* (Mm00438422_m1), *Sost* (Mm00470479_m1), *Ctsk* (Mm00484039_m1), *Tnfrsf11b* (= Opg) (Mm00435454_m1) and *Tnfsf11* (= RANKL) (Mm00441906_m1). *B2m* (Mm00437762_m1) and *GAPDH* (Mm99999915_g1) were used for normalization (averaged). Relative quantification was calculated using the ΔCt method.

### Statistical analysis

The within-subject effect of loading (loaded and control limbs) and between-subject effects of age (10- and 26-week-old) and genotype (LC and *Sost* KO) as well as interactions between these terms were assessed using a repeated measure ANOVA (SAS 9.3, Cary, USA). A separate ANOVA was used to assess between-subject age (10- and 26-week-old) and genotype (LC and *Sost* KO) and interaction effects for relative values, the interlimb differences (∆_interlimb_ = loaded limb − control limb). Paired t-tests were used to compare control and loaded limbs. Unpaired t-tests were performed to compare control limbs between age or genotype. The percent difference was presented as percentual increment [%Δ = ((loaded limb − control limb)/control limb) × 100%)]. Statistical analyses of the qPCR data were performed on the ΔCt values. Unpaired or paired t-tests were performed to determine the differences between *Sost* KO and LC or between loaded and control limbs, respectively. A significant difference was set for all analyses as *p* < 0.05.

## Results

### Tissue strains in cortical bone of Sost KO and age- and gauge strain-matched LC mice

**Figure 2:**
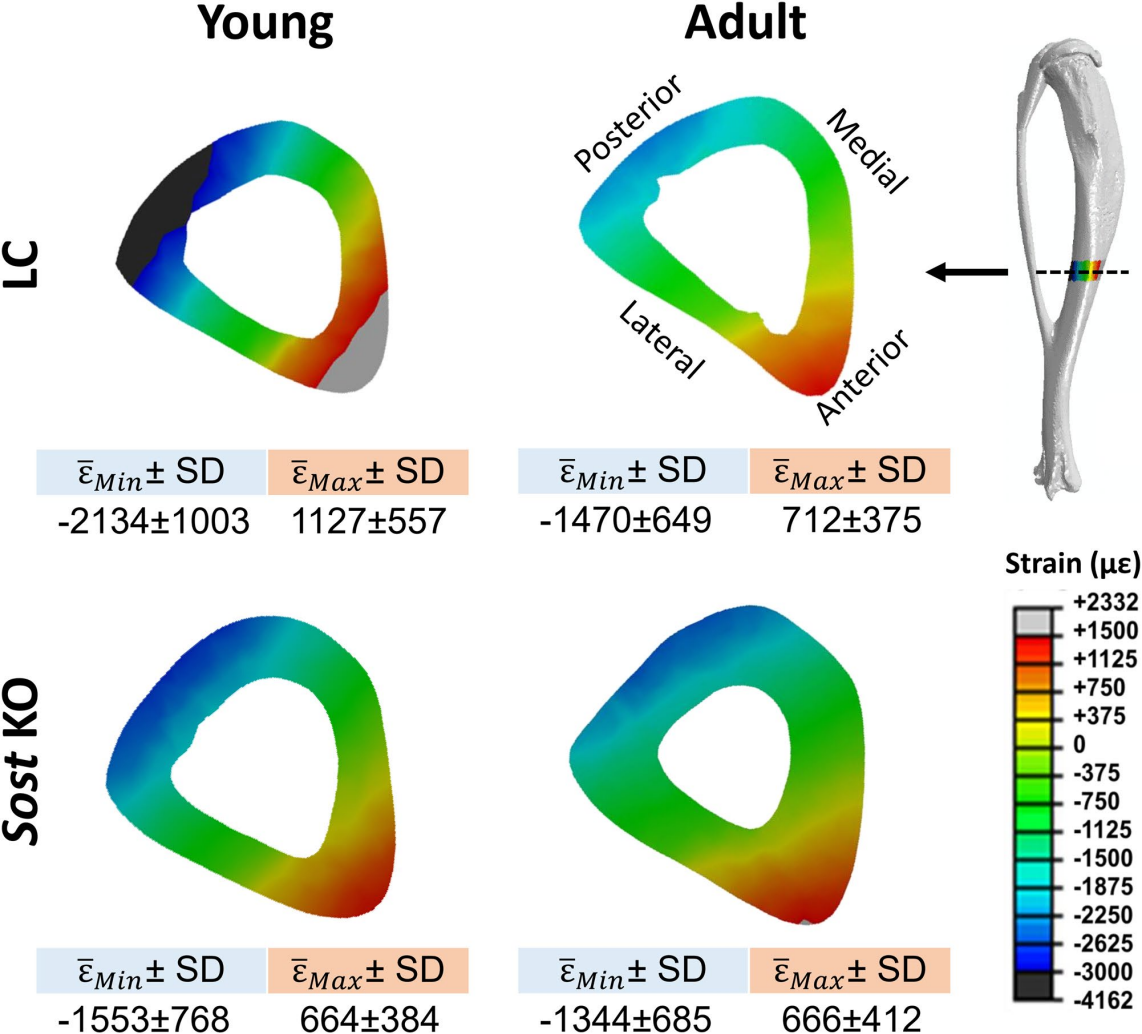
In vivo load-induced strain distribution in the midshaft cortical bone of the tibiae of young (10-week-old) and adult (26-week-old) littermate control (LC) and *Sost* knockout (KO) male mice. Red and blue indicate tension and compression, respectively.

The strain values at the strain gauge site predicted by the FE models were similar to those measured experimentally (~ 900 µε), supporting the validation of our FE models. Further, the FE models predicted that the mean compressive strains were 16% higher in 10-week-old than in 26-week-old *Sost* KO mice (Fig. 2). In contrast, the mean tensile strains were almost identical in the 10- and 26-week-old *Sost* KO mice. Both mean tensile and compressive strains were lower in the *Sost* KO mice than in their age-matched LC whereas this difference was much more pronounced in the 10-week-old (37% for tension and 70% for compression) than in the 26-week-old mice (7% for tension and 9% for compression) (Fig. 2).

### Sost deficiency led to an increased bone formation in male mice

**Figure 3:**
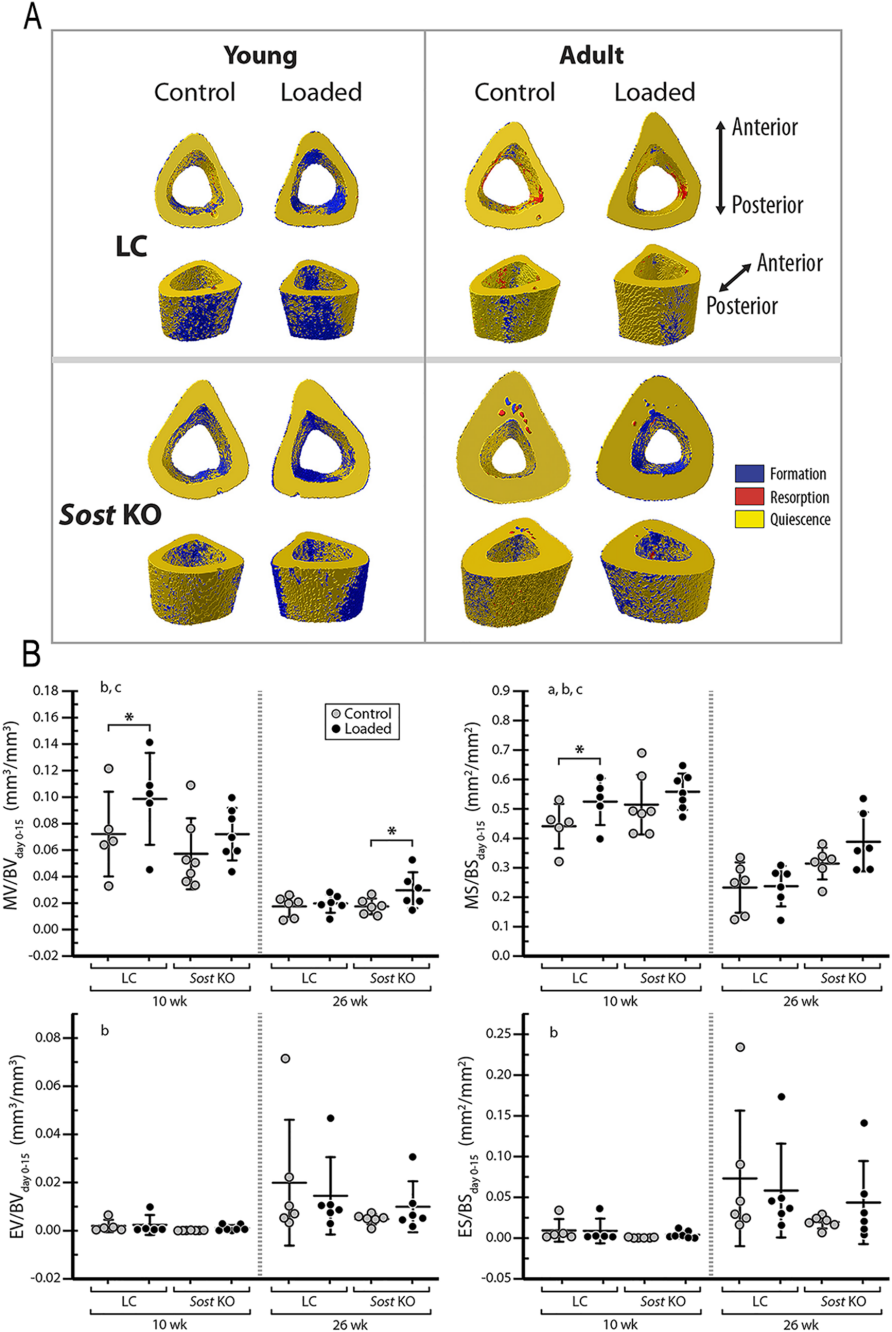
(**A**) Visualization of newly formed (blue), resorbed (red), and quiescent (yellow) bone tissues over 15 days at the midshaft of control and loaded tibiae from young (10-week-old) and adult (26-week-old) LC and *Sost* KO male mice measured with 3D dynamic time-lapse in vivo morphometry. (**B**) MV/BV_day0–15_ or MS/BS_day0–15_ indicate the amounts of newly formed bone volume or surface area between day 0 and 15 (the loading period) relative to the total bone volume or surface at day 0. EV/BV_day0–15_ or ES/BS_day0–15_ indicate the amounts of resorbed bone volume or surface area between day 0 and 15 relative to the total bone volume or surface at day 0. ANOVA: indicates an effect of (a) genotype, (b) age, (c) loading, (d) genotype and age, (e) genotype and loading, (f) age & loading. *p < 0.05 by paired t-test. Sample size: 10-week-old LC (n = 5), 10-week-old *Sost* KO (n = 7), 26-week-old LC (n = 6), 26-week-old *Sost* KO (n = 6).

**Figure 4:**
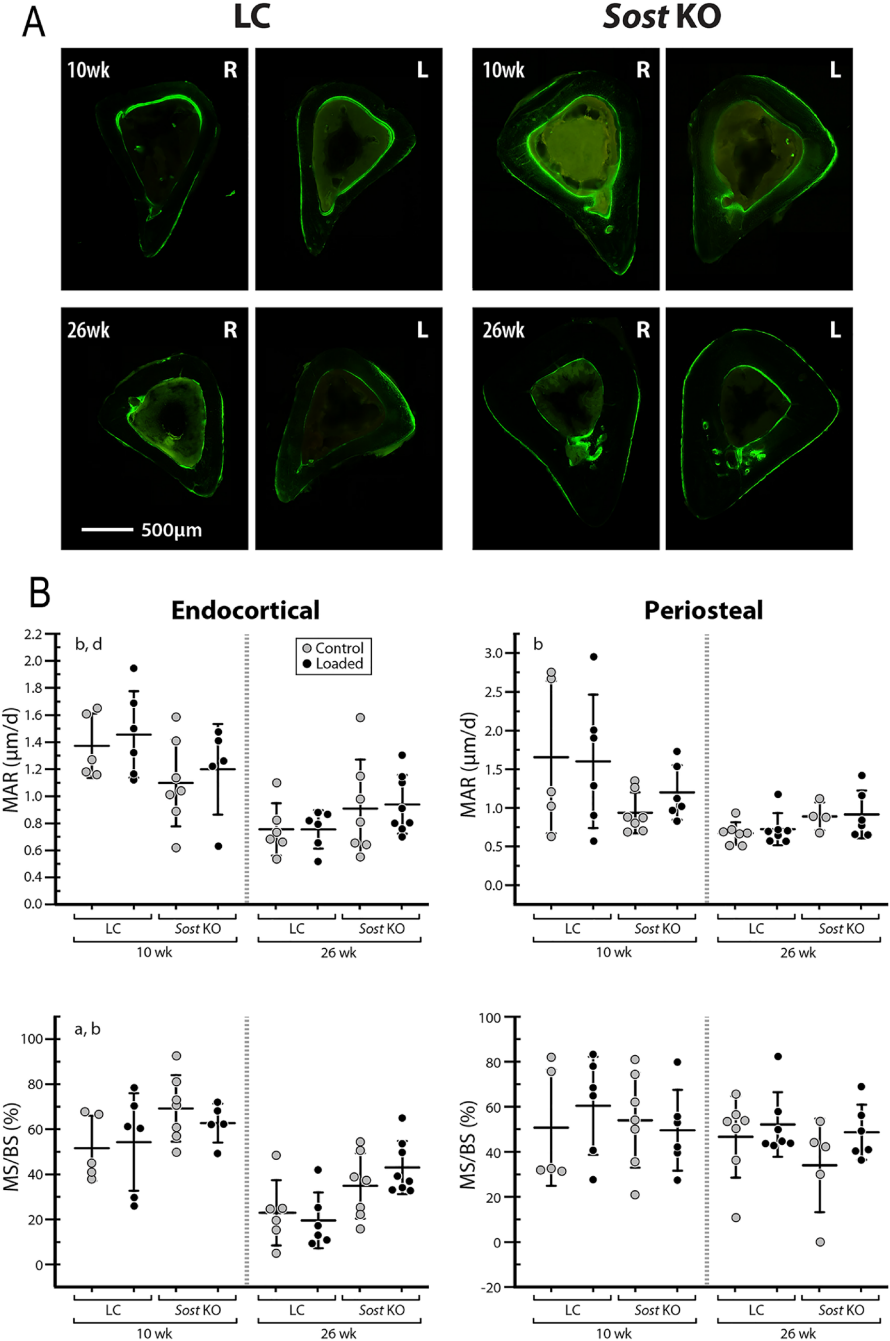
Cortical histomorphometry: (**A**) cross-sectional images showing fluorochrome labeling of the midshaft cortical bone at day 3 and 12; (**B**) mineral apposition rate (MAR) and mineralizing surface normalized to bone surface (MS/BS) are shown for both the endocortical and periosteal surfaces. MAR is the distance between the labels divided by time between labels. MS/BS indicates the extent of bone surface actively mineralizing. ANOVA: indicates an effect of (a) genotype, (b) age, (c) loading, (d) genotype & age, (e) genotype & loading, (f) age & loading. Sample size: 10-week-old LC (n = 5–6), 10-week-old *Sost* KO (n = 5–7), 26-week-old LC (n = 6–7), 26-week-old *Sost* KO (n = 4–8).

Compared to LC mice, male *Sost* KO mice had a greater surface area of newly mineralized tissues relative to the total surface area at the midshaft cortical bone, as indicated by time-lapse in vivo morphometry (main effect of genotype for MS/BS_day0–15_; Fig. 3). Further, both in vivo morphometry and histomorphometry showed that *Sost* deficiency-induced increase in MS/BS occurred primarily at the endocortical surfaces (main effect of genotype for Ec.MS/BS and Ec. MS/BS_day0–15_; Fig. 4, Supplementary Tables 3 and 4). Consistent with the enhancement of bone formation due to *Sost* deficiency, 10- and 26-week-old *Sost* KO mice had greater morphological and mechanical parameters (Ct.Ar, T.Ar, Ct.Th, I_max_ and I_min_) relative to their littermate controls (main effects of genotype; Table 1). Additionally, those microCT-measured parameters were enhanced in 26-week-old *Sost* KO mice compared to 10-week-old mice (interaction of age and genotype; Table 1), likely due to an accumulation of formed bone associated with long-term *Sost* deficiency. *Sost* deficiency had little effect on bone resorption (no main effect of genotype; Fig. 3B).

**Table 1:** Cortical bone parameters of the tibial midshaft, measured from in vivo microCT at day 0, 5, 10 and 15, in 10- and 26-week-old LC and *Sost* KO male mice exposed to axial tibial compression (left tibia loaded, right tibia nonloaded control).

Parameters	10-week-old				26-week-old			
	LC		*Sost* KO		LC		*Sost* KO	
	Control	Loaded	Control	Loaded	Control	Loaded	Control	Loaded
**Day 0**								
Imax (mm^4^)	0.07 ± 0.01	0.06 ± 0.01	0.13 ± 0.02	0.12 ± 0.02	0.10 ± 0.01	0.10 ± 0.02	0.27 ± 0.05	0.25 ± 0.05
Imin (mm^4^)	0.05 ± 0.01	0.05 ± 0.01	0.10 ± 0.01	0.09 ± 0.01	0.07 ± 0.01	0.07 ± 0.01	0.20 ± 0.03	0.19 ± 0.03
Ct.Ar (mm^2^)	0.52 ± 0.05	0.50 ± 0.05	0.81 ± 0.08	0.81 ± 0.06	0.67 ± 0.05	0.67 ± 0.05	1.39 ± 0.12	1.35 ± 0.13
T.Ar (mm^2^)	0.99 ± 0.05	0.95 ± 0.07	1.32 ± 0.08	1.24 ± 0.09	1.16 ± 0.10	1.15 ± 0.11	1.78 ± 0.14	1.73 ± 0.14
Ct.Ar/T.Ar (mm^2^/mm^2^)	0.53 ± 0.03	0.53 ± 0.02	0.62 ± 0.04	0.66 ± 0.02	0.58 ± 0.02	0.58 ± 0.03	0.78 ± 0.02	0.78 ± 0.03
Ct.Th (µm)	176 ± 15	172 ± 13	247 ± 22	255 ± 13	211 ± 9	209 ± 11	378 ± 23	357 ± 51
Ct.TMD (mg HA/cm^3^)	1280 ± 21	1235 ± 11	1262 ± 24	1263 ± 29	1332 ± 12	1319 ± 20	1374 ± 19	1349 ± 43
**Day 5**								
Imax (mm^4^)	0.08 ± 0.01	0.07 ± 0.01	0.14 ± 0.02	0.13 ± 0.02	0.11 ± 0.02	0.10 ± 0.02	0.29 ± 0.06	0.27 ± 0.05
Imin (mm^4^)	0.06 ± 0.01	0.05 ± 0.01	0.10 ± 0.01	0.10 ± 0.01	0.07 ± 0.02	0.07 ± 0.01	0.20 ± 0.03	0.20 ± 0.03
Ct.Ar (mm^2^)	0.56 ± 0.05	0.53 ± 0.06	0.84 ± 0.07	0.84 ± 0.06	0.68 ± 0.06	0.66 ± 0.04	1.41 ± 0.11	1.37 ± 0.11
T.Ar (mm^2^)	1.05 ± 0.05	0.98 ± 0.08	1.33 ± 0.07	1.26 ± 0.06	1.17 ± 0.12	1.16 ± 0.11	1.82 ± 0.15	1.77 ± 0.13
Ct.Ar/T.Ar (mm^2^/mm^2^)	0.53 ± 0.02	0.54 ± 0.02	0.63 ± 0.03	0.66 ± 0.02	0.58 ± 0.03	0.58 ± 0.03	0.77 ± 0.03	0.77 ± 0.03
Ct.Th (µm)	179 ± 14	176 ± 13	255 ± 19	262 ± 17	210 ± 10	209 ± 8	373 ± 27	364 ± 31
Ct.TMD (mg HA/cm^3^)	1275 ± 11	1230 ± 20	1260 ± 26	1263 ± 34	1336 ± 31	1316 ± 21	1371 ± 16	1316 ± 30
**Day 10**								
Imax (mm^4^)	0.08 ± 0.02	0.07 ± 0.01	0.14 ± 0.02	0.13 ± 0.02	0.11 ± 0.02	0.10 ± 0.02	0.29 ± 0.04	0.26 ± 0.05
Imin (mm^4^)	0.06 ± 0.01	0.05 ± 0.01	0.10 ± 0.01	0.10 ± 0.01	0.07 ± 0.01	0.07 ± 0.01	0.20 ± 0.03	0.20 ± 0.03
Ct.Ar (mm^2^)	0.57 ± 0.07	0.56 ± 0.06	0.87 ± 0.07	0.86 ± 0.06	0.68 ± 0.05	0.67 ± 0.04	1.40 ± 0.10	1.37 ± 0.11
T.Ar (mm^2^)	1.05 ± 0.08	1.00 ± 0.09	1.36 ± 0.07	1.31 ± 0.10	1.19 ± 0.12	1.17 ± 0.11	1.83 ± 0.12	1.74 ± 0.15
Ct.Ar/T.Ar (mm^2^/mm^2^)	0.54 ± 0.03	0.56 ± 0.02	0.64 ± 0.03	0.66 ± 0.05	0.57 ± 0.03	0.58 ± 0.03	0.77 ± 0.02	0.79 ± 0.03
Ct.Th (µm)	187 ± 17	186 ± 12	260 ± 22	265 ± 25	210 ± 7	210 ± 9	368 ± 30	377 ± 33
Ct.TMD (mg HA/cm^3^)	1281 ± 12	1243 ± 29	1284 ± 12	1263 ± 21	1341 ± 13	1315 ± 25	1351 ± 27	1348 ± 24
**Day 15**								
Imax (mm^4^)^a,b,d^	0.09 ± 0.01	0.08 ± 00.2	0.15 ± 0.02	0.14 ± 0.01	0.12 ± 0.03	0.11 ± 0.03	0.29 ± 0.04	0.26 ± 0.04
Imin (mm^4^)^a,b,d^	0.06 ± 0.02	0.06 ± 0.01	0.11 ± 0.01	0.11 ± 0.01	0.08 ± 0.02	0.07 ± 0.01	0.21 ± 0.03	0.20 ± 0.02
Ct.Ar (mm^2^)^a,b,d^	0.60 ± 0.07	0.58 ± 0.07	0.89 ± 0.08	0.89 ± 0.06	0.69 ± 0.06	0.68 ± 0.05	1.42 ± 0.11	1.39 ± 0.10
T.Ar (mm^2^)^a,b,d^	1.09 ± 0.08	1.03 ± 0.11	1.40 ± 0.06	1.33 ± 0.06	1.21 ± 0.13	1.19 ± 0.12	1.83 ± 0.11	1.76 ± 0.11
Ct.Ar/T.Ar (mm^2^/mm^2^)^a,b,c,d^	0.55 ± 0.03	0.56 ± 0.02	0.64 ± 0.04	0.67 ± 0.04	0.57 ± 0.03	0.57 ± 0.04	0.77 ± 0.02	0.79 ± 0.04
Ct.Th (µm)^a,b,d^	189 ± 20	192 ± 13	263 ± 26	272 ± 23	204 ± 13	200 ± 19	373 ± 29	375 ± 31
Ct.TMD (mgHA/cm^3^)^b,d^	1293 ± 15	1253 ± 27	1273 ± 20	1262 ± 17	1352 ± 33	1296 ± 45	1374 ± 20	1346 ± 18

Data are given as mean ± SD. ANOVA: indicates an effect of (**a**) genotype, (**b**) age, (**c**) loading, (**d**) genotype & age, (**e**) genotype & loading, (**f**) age & loading, *p* < 0.05.

### Skeletal maturation led to decreased bone formation and increased bone resorption in male Sost KO mice

Skeletal maturation led to decreased bone formation apparent in time-lapse in vivo morphometry, and histomorphometry (main effect of age by ANOVA; Figs. 3 and 4). Time-lapse in vivo morphometry showed decreased MV/BV_day0–15_ and MS/BS_day0–15_ in 26-week-old compared to 10-week-old *Sost* KO and LC mice (Fig. 3B). Histomorphometry measures also revealed a decrease in bone formation with skeletal maturation (Ec.dLS/BS, Ec.MS/BS, Ec.MAR, Ec.BFR/BS, Ps.sLS/BS, Ps.dLS/BS, Ps. MAR) (Fig. 4 and Supplementary Table 4). Interestingly, this reduction in bone formation with skeletal maturation was independent of *Sost* deficiency except in the Ec.MAR (ANOVA, interaction of genotype and age; Fig. 4B). Skeletal maturation also affected bone resorption, with 26-week-old mice having larger EV/BV_day0–15_ and ES/BS_day0–15_ than 10-week-old mice (Fig. 3B).

### The moderate loading level led to only a mild anabolic response in male Sost KO mice

**Figure 5:**
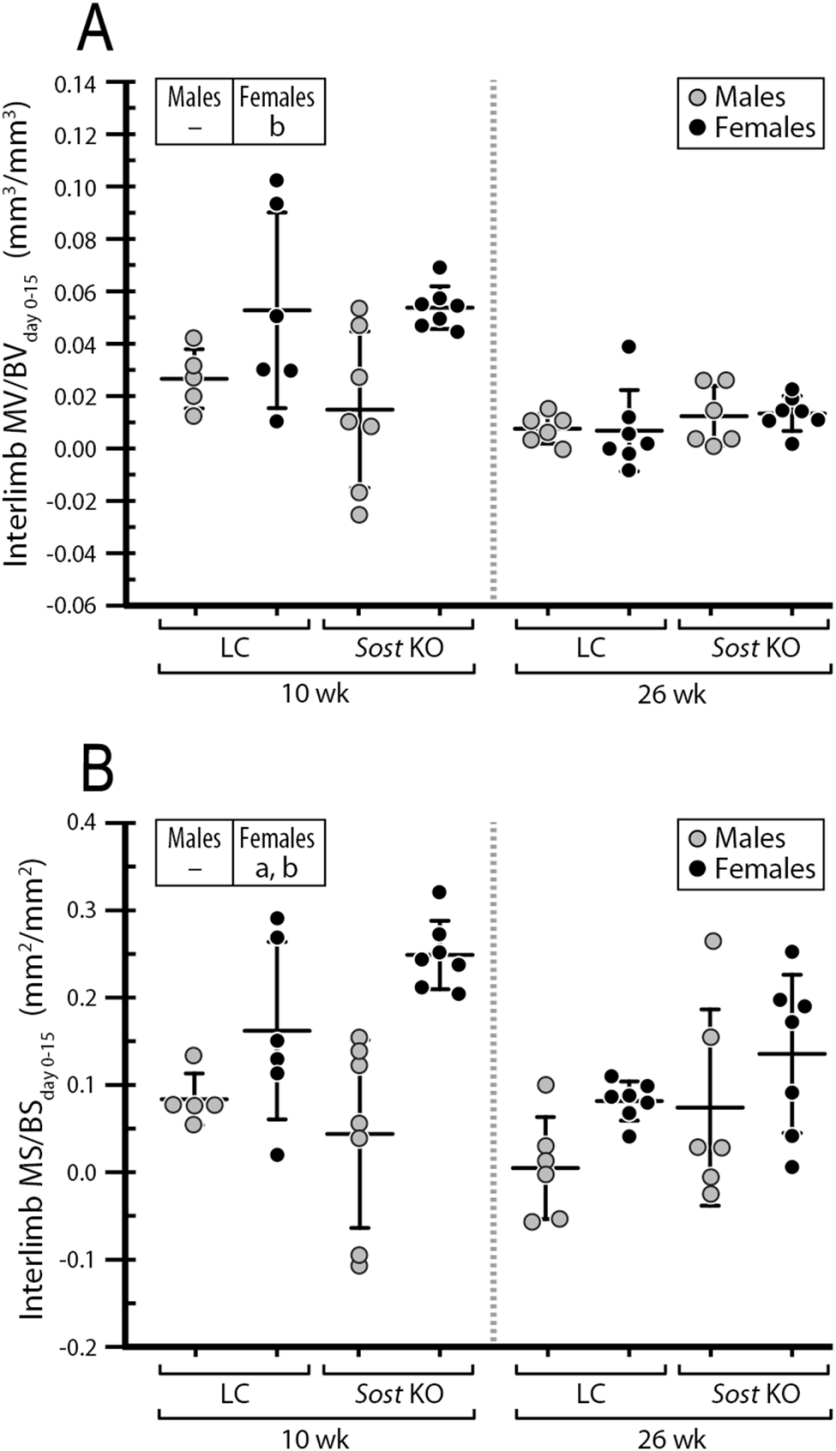
Time-lapse in vivo morphometry measured newly formed bone between day 0 and day 15, including the total mineralizing volume (MV) and surface area (MS) normalized to the total bone volume (BV) or bone surface (BS) at day 0. Data is shown as interlimb difference (loaded-control limb) comparing male with previously published data for female mice^10^. ANOVA: indicates an effect of (a) genotype, (b) age, (c) genotype & age, which was performed separately for each sex. Sample size: 10-week-old LC (male = 5, female = 6), 10-week-old *Sost* KO (male = 7, female = 7), 26-week-old LC (male = 6, female = 7), 26-week-old *Sost* KO (male = 6, female = 7).

A significant main effect of loading was observed in MV/BV_day0–15_ and MS/BS_day0–15_) (ANOVA; Fig. 3 and Supplementary Table 3). Sub-analyses indicated that, in 26-week-old *Sost* KO mice, the loaded limb had a significantly greater MV/BV_day0–15_ (+ 76%) than its contralateral limb (*p* < 0.05 by paired t-test; Fig. 3B). Compared to the control limb, the loaded limb of the 10-week-old LC mice had significantly increased MV/BV_day0–15_ (+ 38%) and MS/BS_day0–15_ (+ 18%) (*p* < 0.05 by paired t-test; Fig. 3B), which occurred primarily at the endocortical surfaces (Ec.MV/BV_day0–15_: + 118%; Ec.MS/BS_day0–15_: + 100%; Supplementary Table 3). Additionally, loading had no effect on EV/BV or ES/BS (Fig. 3B). Although a main effect of loading on bone formation response to loading was observed, there was no interactive effect between genotype and loading (ANOVA; Fig. 3B). This was further confirmed in that there was no significant effect of genotype on the interlimb differences (loaded limb – control limb) in MV/BV_day0–15_ or MS/BS_day0–15_ for males (Fig. 5). Consistent with the observed load-induced bone formation at the endocortical surfaces by in vivo morphometry, static microCT measures at day 15 showed a significant load-induced increase in the cortical area fraction (Ct.Ar/T.Ar) (Table 1). However, bone formation indices from histomorphometry failed to show any loading effect (Fig. 4 and Supplementary Table 4).

### Gene expression was altered in male Sost KO mice

**Figure 6:**
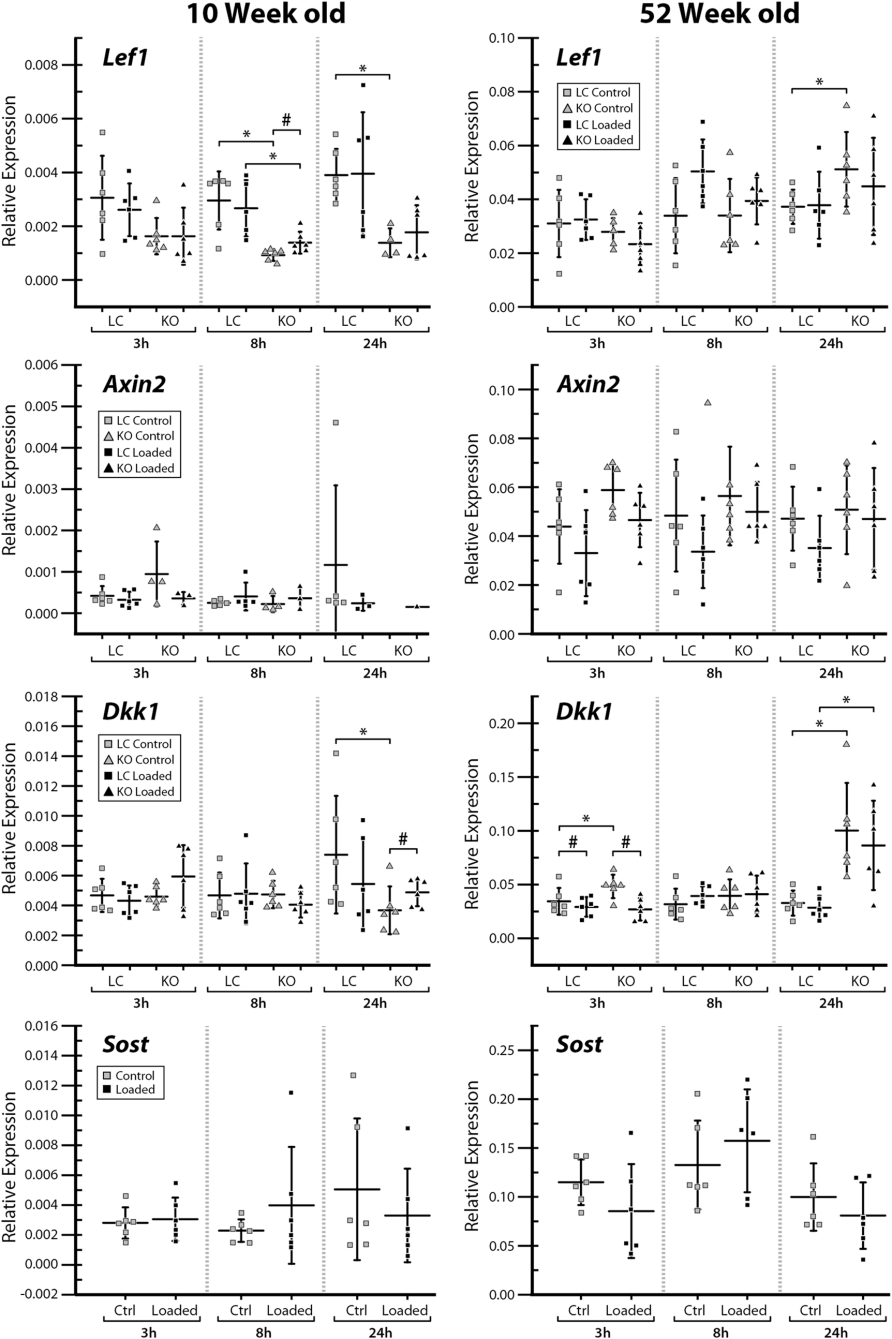
Gene expression of *Lef1*, *Axin2*, *Dkk1* and *Sost* was measured at 3, 8, and 24 h after a single loading session in the left loaded and right control tibiae of 10- and 52-week-old male *Sost* KO and LC mice. Gene expressions relative to reference genes are shown. *Indicates a significant difference between *Sost* KO compared to LC for each condition (t-test; *p* < 0.05). # Indicates a significant difference between loaded and control bones for each condition (paired t-test; *p* < 0.05). Sample size: n = 3–6.

**Figure 7:**
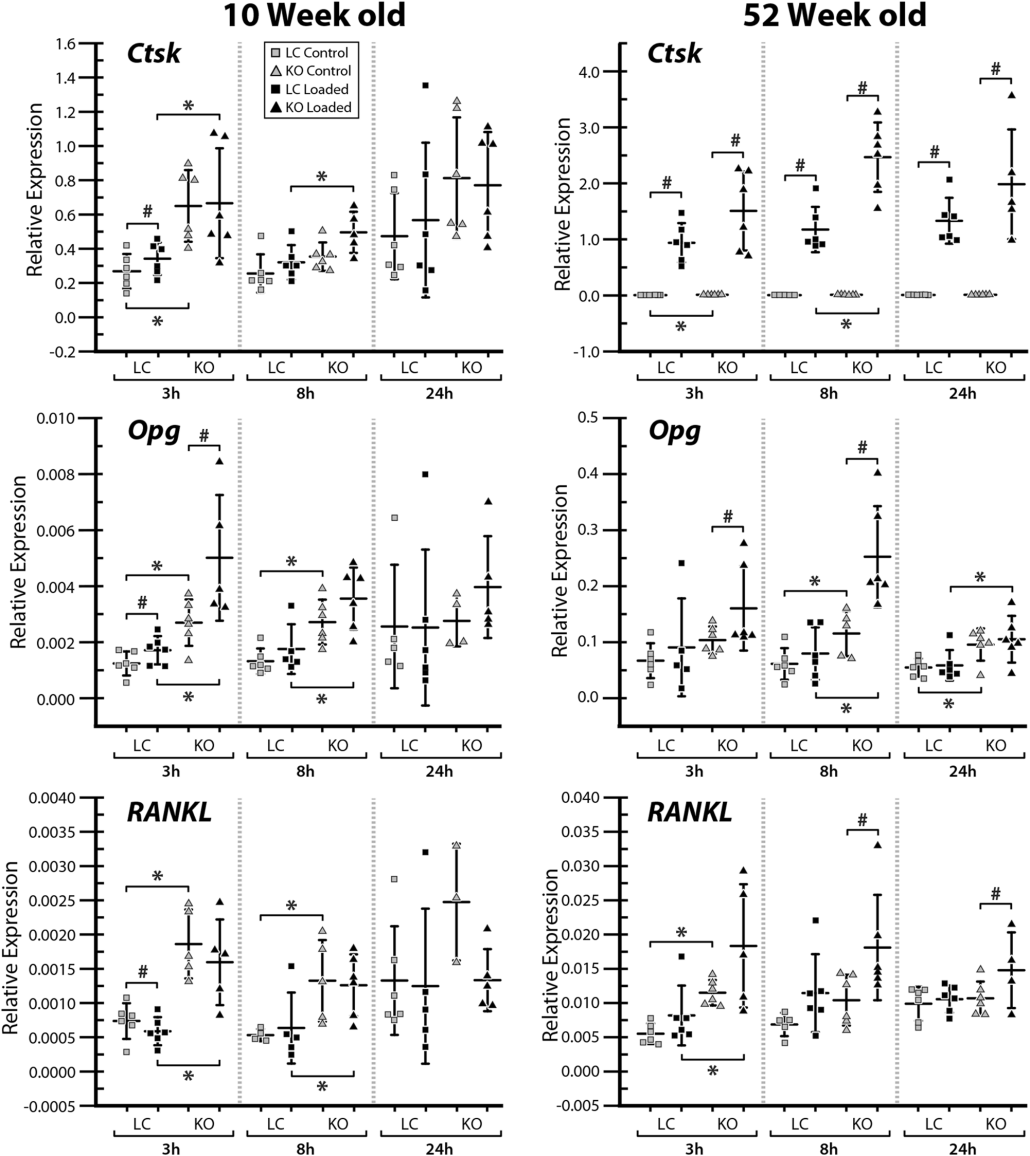
Gene expression of *Ctsk*, *Tnfrsf11b* (= *Opg*) and *Tnfsf11* (= *RANKL*) was measured at 3, 8, and 24 h after a single loading session in the left loaded and right control tibiae of 10- and 52-week-old male *Sost* KO and LC mice. Gene expressions relative to reference genes are shown. *Indicates a significant difference between *Sost* KO compared to LC for each condition (t-test; *p* < 0.05). # Indicates a significant difference between loaded and control bones for each condition (paired t-test; *p* < 0.05). Sample size: n = 3–6.

Ten-week-old *Sost* KO mice had decreased *Lef1* expression in both loaded and control limbs compared to LC mice, which was significant at 8 h and 24 h and approaching significance at 3 h (Fig. 6). In contrast, *Sost* deficiency led to increased *Lef1* expression at 24 h in 52-week-old mice (Fig. 6). We did not detect changes in *Axin2* expression levels. *Dkk1* was significantly increased in 52-week-old *Sost* KO mice at 3 h and 24 h compared to LC mice. In contrast, we detected lower expression of *Dkk1* in control limbs of 10-week-old *Sost* KO at the 24 h time point (Fig. 6). *Ctsk*, Opg and RANKL mRNA levels were higher in 10-week-old *Sost* KO vs LC mice in many conditions at 3 h and 8 h, but not 24 h after loading (Fig. 7). In 52-week-old males, *Ctsk* was increased in *Sost* KO mice 3 h after loading in nonloaded limbs and 8 h after loading in loaded limbs; *Sost* depletion increased Opg expression in both loaded and control limbs at the 8 h and 24 h time points (Fig. 7). RANKL expression was increased in control and loaded limbs of 52-week-old *Sost* KO mice only 3 h after loading (Fig. 7).

The *Wnt* effector gene *Lef1* was increased in the loaded limb of 10-week-old *Sost* KO mice at 8 h, but the effect was abrogated at 24 h (Fig. 6). Loading did not influence *Lef1* expression in 10- or 52-week-old LC mice or *Axin2* expression in either *Sost* KO or LC mice (Fig. 6). *Dkk1* expression was increased with loading at 24 h in 10-week-old *Sost* KO mice. At 3 h after loading we observed a decrease in the *Wnt* inhibitor *Dkk1* expression in both 52-week-old *Sost* KO and LC mice (Fig. 6). We did not observe a change in *Sost* gene expression in LC mice following loading. There was a slight increase in *Ctsk* expression 3 h after loading in 10-week-old LC mice (Fig. 7). Loading caused dramatic increases in *Ctsk* expression in 52-week-old LC and *Sost* KO mice at 3 h, 8 h and 24 h. We also observed increases in Opg expression after loading in 10-week-old (LC and *Sost* KO, 3 h time point) and 52-week-old (*Sost* KO, 3 h and 8 h) mice. RANKL was affected differently by loading between 10- and 52-week-old mice. Specifically, RANKL expression was decreased at 3 h following loading in 10-week-old LC mice while it was increased at 8 h and 24 h in 52-week-old *Sost* KO mice (Fig. 7).

## Discussion

Motivated by our earlier work on females, the goal of this study was to examine, in male mice, the effect of *Sost* deficiency on the cortical bone (re)modeling and adaptive response to a moderate level of applied loading. In vivo loading was applied to the tibiae of young and older male *Sost* KO and LC mice to engender a gauge strain of ~ 900 με at the midshaft, the same target strain as our previous female loading study^10^. This more moderate target strain level was chosen in the present study in males and previous study in female *Sost* KO mice^10^, since our pilot studies showed that 1200 με (requiring 17 N) determined through in vivo strain gauging of 10-week-old female *Sost* KO mice at the mid-diaphysis led to ankle swelling and limping in the mice during the first several days of loading.

As expected, *Sost* deficiency led to high cortical bone mass in young and adult male mice as a result of increased bone formation. However, the enhanced bone formation associated with *Sost* deficiency did not appear to diminish with skeletal maturation. An increase in bone resorption was observed with skeletal maturation in male LC and *Sost* KO mice. Our finding that gene expression levels for cathepsin K, Opg and RANKL are higher in *Sost* KO males (independent of loading in many conditions) suggests that more bone remodeling might be taking place in the absence of *Sost*. The applied moderate level of loading only induced a mild anabolic response in young and adult male mice, independent of *Sost* deficiency. Loading led to a decrease in the Wnt inhibitor *Dkk1* expression at 3 h in middle-aged *Sost* KO and LC mice, and an increase in *Lef1* expression at 8 h in young *Sost* KO mice. The current results suggest that in terms of bone mass, microstructure, and (re)modeling, long-term inhibition of sclerostin in male mice does not influence the adaptive response of cortical bone to moderate levels of loading.

**Figure 8:**
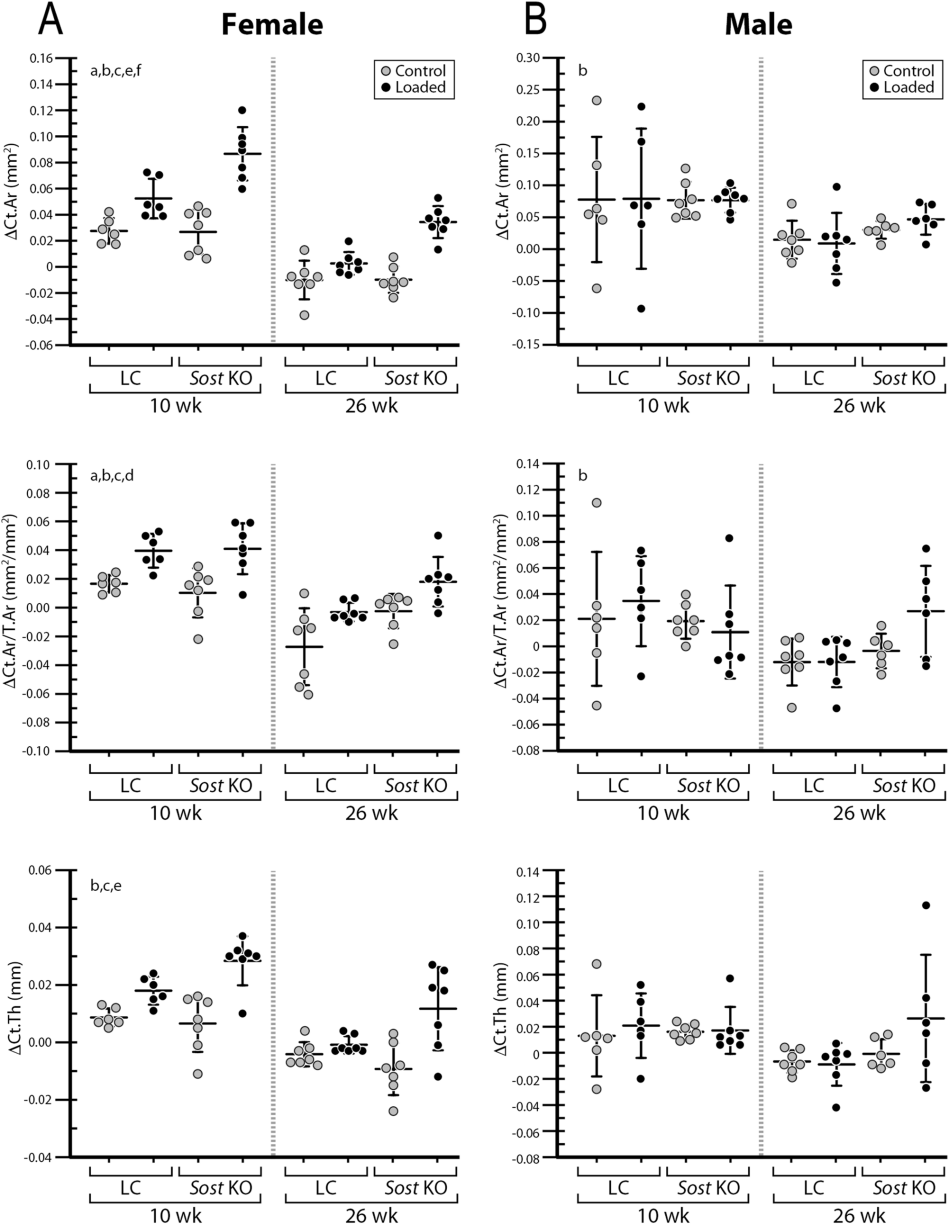
MicroCT results of cortical area (ΔCt.Ar), cortical area divided by total area (ΔCt.Ar/T.Ar) and cortical thickness (ΔCt.Th) by in vivo microCT expressed as (ΔX = X_day15_** − **X_day0_ within the same limb). Previously published data in female 10- and 26-week-old LC and *Sost* KO mice are shown here for comparison with male mice^10^. ANOVA: indicates an effect of (a) genotype, (b) age, (c) loading, (d) genotype and age, (e) genotype and loading, (f) age & loading, *p* < 0.05. Sample size: 10-week-old LC (male = 6, female = 6), 10-week-old *Sost* KO (male = 7, female = 7), 26-week-old LC (male = 7, female = 7), 26-week-old *Sost* KO (male = 6, female = 7).

Although *Sost* deficiency did not affect cortical bone response to loading in the male mice, it significantly enhanced cortical mechanoresponse in the female mice (Figs. 5 and 8)^10^, given the same target strain (~ 900 με) at the tibial midshaft for both males and females. Also, a reduction in bone resorption (ES/BS) in response to loading was previously observed in adult female *Sost* KO mice^10^, while it was not observed in the male *Sost* KO mice. These results suggest that the effect of *Sost* deficiency on cortical bone response to loading may be sex dependent, at least for the examined moderate level of loading which might be associated with moderate-intensity physical activities. Others have reported in 16-week-old female *Sost* KO mice a dose response of cortical bone to incremental loading^13^. Interestingly, they also found that female *Sost* KO mice had lower load-induced bone formation rate (rBFR/BS) relative to wild-type mice in high strain regions, but greater rBFR/BS relative to wild-type mice in low strain regions^13^. It remains unclear whether the effect of *Sost* deficiency on cortical mechanoresponse differs between males and females at a higher level of applied loading than that of the current study. However, a higher load might have less clinical relevance since patients suffering from osteoporosis might not be able to take any high-intensity activities.

In terms of the bone (re)modeling responses to *Sost* deficiency, both male and female *Sost* KO mice showed an elevated bone formation compared to their LCs. However, a reduction in bone resorption associated with *Sost* deficiency was observed in female mice^10^ but not in the male mice (Fig. 3B). This observation is consistent with synchronously elevated RANKL and Opg expressions at 3 and 8 h in the 10-week-old *Sost* KO male mice (Fig. 7). Additionally, skeletal maturation-related increase in bone resorption was diminished in female *Sost* KO mice relative to their LCs, whereas the effect of skeletal maturation on bone resorption was independent of *Sost* deficiency in male mice (Fig. 3B). These results together with those discussed above, suggest that the effect of *Sost* deficiency on cortical bone (re)modeling and adaptation to loading may differ between males and females.

Age‐related impairment of bones’ adaptive response to loading has been consistently shown in female wildtype mice^27,30,32,33,35^, yet the effect of sex remains contradictory across different ages. In addition, little is known regarding the effect of sex on age-related bone mechanoresponse in the absence of *Sost*. In one study examining the effect of ERα in osteoblast-lineage cells on bone mass and adaptation to mechanical loading, 10-week-old male littermate control mice showed less cortical and cancellous bone response compared to females^29^. An additional tibial loading study reported greater diaphyseal cortical and metaphyseal cancellous bone responses to loading in 17-week-old female wild-type mice compared to males of the same age^28^. In 19-month-old aged C57BL/6 mice, however, males and females showed similar cortical anabolic responses to loading^27^. Studies investigating the role of the low-density lipoprotein receptor-related protein 5 (Lrp5) in bones’ responses to loading, showed significant dose response of cortical bone in 17-week-old wild-type male mice but not in females^23^. Taken together, it remains unclear how sex affects bone adaptation to loading with skeletal maturation. Under the moderate loading examined, our current and previous results showed that both male and female LC mice had a reduced mechanoresponse with skeletal maturation, and this reduction appears to be greater in females than in males. Specifically, loading led to significant increases in the amount of newly mineralized bone volume and mineralizing surface area (MV/BV_day0–15_: + 38%, MS/BS_day0–15_: + 18%) in the 10-week-old LC male mice. However, no load-induced change in MV/BV_day0–15_ and MS/BS_day0–15_ was observed in 10-week-old LC male mice. We also observed a reduction of cortical bone formation response to loading with skeletal maturation in female *Sost* KO mice^10^ but not in male *Sost* KO mice (no interactive effect between age and loading was found by ANOVA for MV/BV_day0–15_ and MS/BS_day0–15_ in 10- and 26-week-old male *Sost* KO mice).

Similar to our previous study in female mice^10^, we observed decreased *Lef1* in young male *Sost* KO mice compared to LC mice. A lower expression of *Lef1* was measured in the loaded limbs of young female *Sost* KO mice at 8 h and adult *Sost* KO mice at 8 and 24 h compared to LC mice. Lin et al. also observed this interaction between genotype and age^54^, however, they reported greater *Lef1* expression in young *Sost* KO mice compared to wild-type mice, but no difference in adult mice. We observed increased *Dkk1* expression in middle-aged male *Sost* KO compared to LC mice, which was similar to young and adult *Sost* KO female mice^10^, which had increased *Dkk1* expression compared to LC mice. Increased expression of this other Wnt inhibitor, *Dkk1*, may be due to a feedback mechanism intended to compensate for the loss of *Sost*. We did not observe a significant decrease in *Sost* with loading. This could be because male mice might have a greater adaptive strain threshold than females (see discussion below). Others have also reported a lack of downregulation in *Sost* after loading^55^. Recent unpublished data from our group shows that the time of day at which the mouse is loaded alters *Sost* expression due to circadian rhythms, which might explain our finding^56^. Future research is required to have a deeper understanding of the molecular mechanisms for the sex-dependent bone mechanoresponse due to sclerostin inhibition.

In general, the increases in load-induced bone formation for both *Sost* KO and LC mice were greater in females compared to males. One possible explanation for the dampened loading response in males could be the group housing of our male mice. A previous study demonstrated that group-housed males engaged in frequent fighting and had an attenuated response to mechanical loading compared with single-housed males^26^. However, since our mice are littermates, we expected limited fighting and only observed fighting on one occasion, whereby the mice were immediately separated. Another explanation could be a difference in strain threshold for stimulating new bone formation in male and females^27^. Since age-matched male mice are generally heavier than females (Supplementary Table 2), they may experience greater habitual loading during natural cage activities. The strain threshold already derived from habitual loading^41^ might be higher for males than females. The mild anabolic response to loading in males relative to females was not due to lower tissue strains. Comparing our FE analysis of male tibiae (Fig. 2), with previous FE results from females^39^, further demonstrates that the average and peak strains in the tibial midshaft VOI of males were greater than in female mice.

There are few limitations. Firstly, although we observed the anabolic response to loading in our time-lapse analyses, we did not observe differences between loaded and control limbs in many microCT-based morphological parameters or bone formation indices measured by histomorphometry. The reason for this discrepancy between methods might be attributed to the fact that the time-lapse analysis looks at the exact same anatomic VOI of bone, since we analyzed the registered (overlapping volume) from the initial and subsequent microCT images. In contrast, the static microCT measures might not examine the exact same VOI of bone, but rather the same relative position. This is a particular concern with longitudinal growth in the long bones, since often only a part of the tibia is scanned during in vivo microCT, to minimize radiation exposure^30^. Also, we have shown how histomorphometry results may depend on the position of the 2D section examined^49^. Time-lapse in vivo 3D morphometry overcomes these limitations via spatial registration of consecutive images and identify relatively small bone adaptive changes under a moderate level of loads. Secondly, we did not examine bone morphometric parameters in middle-aged or elderly mice, which may be more clinically relevant for osteoporosis studies, although sclerostin inhibition is also being investigated for use in children with rare bone diseases such as osteogenesis imperfecta. We did not examine gene expression in adult mice, but rather focused on young and middle-aged mice. Two-week loading studies in middle-aged and elderly *Sost* KO mice were not possible since loading levels required to engender 900 µε or greater would have likely damaged the ankle joint, which we observed in prior studies using 17 N to induce 1200 µε in 10-week-old female *Sost* KO mice^10^. Thirdly, the mild (re)modeling response to loading observed in our male *Sost* KO and LC mice is likely in part due to the use of a lower strain of 900 µɛ, which is still higher than strain levels engendered in wild-type C57BL6 mice during normal walking (200–600 µɛ)^41,42^. We have previously observed a much greater formation and anti-resorptive response to loading in C57BL6 mice when engendering 1200 µɛ^30^. However, using a higher strain level was not possible due to concerns related to overloading the mouse’s ankle joints. Regardless, comparisons between males and females were based on the same load-induced target strain at the midshaft VOI. Furthermore, the peak strains in the midshaft cortical volume of interest, determined by finite element analysis (Fig. 2), were much greater than those peak strain values that have been reported to be osteogenic for male and female animals (~ 1000 µɛ)^41,57,58^. More importantly, application of a target strain of 900 µɛ in male mice ensures the consistency with our previous female loading study, based on which comparisons between males and females were performed. Lastly, our previous study in females was completed shortly before we began our studies in males. However, a direct comparison at the same time would have been preferable, but we did use the same target strain and methodology including the same global threshold for microCT analysis between the studies. Repeating our results in another set of female mice was not allowed by our animal use committee.

In summary, we investigated the effect of *Sost* deficiency and skeletal maturation in male mice on cortical bone morphology, cortical bone (re)modeling and gene expression as well as the local mechanical strain environment induced within the bone under tibial loading. Our key findings were: (1) a moderate level of loading led to modest anabolic response in male mice, independent of *Sost*; (2) *Lef1* expression was significantly reduced in 10-week-old *Sost* KO mice compared to littermates and *Dkk1* expression was increased in 52-week-old male *Sost* KO mice compared to LC mice; (3) Comparing the findings in the current study with those from our previous study in females^10^ indicates that *Sost* deficiency influenced the bone formation response to loading not only in an age-dependent, but also in a sex-dependent manner.

## Supplementary information

Supplementary Information
