# Supplementary material for: Cortical bone adaptation to a moderate level of mechanical loading in male Sost deficient mice
Source: Sci Rep. 2020 Dec 18;10:22299. doi: 10.1038/s41598-020-79098-0 (PMC7749116; doi:10.1038/s41598-020-79098-0)
Supplement: Supplementary file 1 — Supplementary Information [file 41598_2020_79098_MOESM1_ESM.docx]

## Cortical bone adaptation to a moderate level of mechanical loading in male *Sost* deficient mice

Haisheng Yang^1^*, Alexander Büttner^2^*, Laia Albiol^2^, Catherine Julien^3^, Tobias Thiele^2^, Christine Figge^2^, Ina Kramer^4^, Michaela Kneissel^4^, Georg N. Duda^2^, Sara Checa^2^, Bettina M. Willie^3^

(*shared authorship)

*^1^Department of Biomedical Engineering, Faculty of Environment and Life,* Beijing University of Technology, Beijing, China

*^2^Julius Wolff Institute, Charité - Universitätsmedizin Berlin, Berlin Germany*

*^3^Research Centre, Shriners Hospital for Children-Canada, Department of Pediatric Surgery, McGill University, Montreal, Quebec, Canada*

*^4^Novartis Institutes for BioMedical Research, Basel, Switzerland*

**Supplementary Table 1:** Peak applied compressive loads engendering 900 με on the medial surface of the tibial midshaft of 10-, 26-, and 52-week-old LC and *Sost* KO male and female mice. Note compressive loads for female mice reported in a previous study are shown here for comparison with males ^(11)^.

|  | **10-week-old** | | **26-week-old** | | **52-week-old** | |
| --- | --- | --- | --- | --- | --- | --- |
|  | **LC** | ***Sost* KO** | **LC** | ***Sost* KO** | **LC** | ***Sost* KO** |
| **Male** |  |  |  |  |  |  |
| Applied load (N) | -7.5 | -10.7 | -9.0 | -14.8 | -9.0 | -18 |
| **Female** |  |  |  |  |  |  |
| Applied load (N) | -7.0 | -12.9 | -7.0 | -14.5 | -9.4 | -18 |

**Supplementary Table 2:** Tibial bone length measured at the end of the two-week loading experiment and body weight measured at the start and end of the loading experiment. Data are presented mean ± SD.

|  | **10-week-old** | | **26-week-old** | |
| --- | --- | --- | --- | --- |
|  | **LC** | ***Sost* KO** | **LC** | ***Sost* KO** |
| **Tibial length** |  |  |  |  |
| Control (mm) | 17.6 ± 0.3 | 15.9 ± 0.4 | 16.6 ± 0.3 | 16.6 ± 0.4 |
| Loaded (mm) | 17.5 ± 0.3 | 16.0 ± 0.3 | 16.4 ± 0.4 | 16.3 ± 0.4 |
| **Body weight** |  |  |  |  |
| Start (g) | 22.5 ± 1.8 | 23.1 ± 1.5 | 27.4 ± 1.9 | 33.0 ± 2.2 |
| End (g) | 24.0 ± 1.9 | 23.4 ± 1.1 | 27.4 ±1.3 | 31.5 ± 2.1 |

**Supplementary Table 3:** Time-lapse in vivo morphometry parameters (mean ± SD) of the cortical bone at the mid-shaft of the male mice. Cortical bone formation and resoption occurring over the fifteen day interval (day 0 to day15) shown on the total surface (endocortical and periosteal) and at each surface. ANOVA results are only shown for total surface (endocortical plus periosteal surface); an effect of (**a**) genotype, (**b**) age, (**c**) loading, (**d**) genotype & age, (**e**) genotype & loading, (**f**) age & loading, p < 0.05.

| **Parameters** | **10-week-old** | | | | **26-week-old** | | | | | | |
| --- | --- | --- | --- | --- | --- | --- | --- | --- | --- | --- | --- |
|  | **LC** | | ***Sost* KO** | | | **LC** | | | | ***Sost* KO** | |
|  | **Control** | **Loaded** | **Control** | **Loaded** | | | | **Control** | **Loaded** | **Control** | **Loaded** |
| **Total Surface** | | |  |  | | |  | |  |  |  |
| MV/BV_day0-15_ (mm³/mm³) ^b, c^ | 0.072 ± 0.032 | 0.099 ± 0.035 | 0.057 ± 0.027 | 0.072 ± 0.02 | | | | 0.017 ± 0.008 | 0.02 ± 0.007 | 0.017 ± 0.006 | 0.03 ± 0.013 |
| MS/BS _day0-15_ (mm²/mm²) ^a,b,c^ | 0.44 ± 0.08 | 0.52 ± 0.08 | 0.51 ± 0.1 | 0.56 ± 0.06 | | | | 0.23 ± 0.09 | 0.24 ± 0.07 | 0.31 ± 0.05 | 0.39 ± 0.1 |
| EV/BV _day0-15_ (mm³/mm³) ^b^ | 0.002 ± 0.003 | 0.002 ± 0.004 | 0 ± 0 | 0.001 ± 0.001 | | | | 0.02 ± 0.026 | 0.014 ± 0.016 | 0.005 ± 0.002 | 0.018 ± 0.021 |
| ES/BS _day0-15_ (mm²/mm²) ^b^ | 0.009 ± 0.014 | 0.009 ± 0.015 | 0.0004 ± 0.0005 | 0.004 ± 0.005 | | | | 0.073 ± 0.083 | 0.058 ± 0.058 | 0.019 ± 0.008 | 0.049 ± 0.051 |
| **Endocortical** |  |  |  |  | | | |  |  |  |  |
| MV/BV _day0-15_ (mm³/mm³) | 0.011 ± 0.008 | 0.024 ± 0.014 | 0.017 ± 0.01 | 0.022 ± 0.01 | | | | 0.001 ± 0.001 | 0.002 ± 0.001 | 0.005 ± 0.003 | 0.008 ± 0.004 |
| MS/BS _day0-15_ (mm²/mm²) | 0.14 ± 0.08 | 0.28 ± 0.14 | 0.32 ± 0.19 | 0.37 ± 0.16 | | | | 0.02 ± 0.02 | 0.03 ± 0.01 | 0.12 ± 0.06 | 0.2 ± 0.12 |
| EV/BV _day0-15_ (mm³/mm³) | 0.002 ± 0.002 | 0.002 ± 0.004 | 0 ± 0 | 0.001 ± 0.001 | | | | 0.02 ± 0.026 | 0.011 ± 0.008 | 0.005 ± 0.002 | 0.014 ± 0.015 |
| ES/BS _day0-15_ (mm²/mm²) | 0.026 ± 0.039 | 0.024 ± 0.045 | 0.002 ± 0.002 | 0.013 ± 0.014 | | | | 0.22 ± 0.235 | 0.138 ± 0.076 | 0.065 ± 0.026 | 0.112 ± 0.077 |
| **Periosteal** |  |  |  |  | | | |  |  |  |  |
| MV/BV _day0-15_ (mm³/mm³) | 0.029 ± 0.018 | 0.039 ± 0.021 | 0.016 ± 0.016 | 0.027 ± 0.012 | | | | 0.003 ± 0.002 | 0.005 ± 0.004 | 0.003 ± 0.002 | 0.009 ± 0.009 |
| MS/BS _day0-15_ (mm²/mm) | 0.3 ± 0.21 | 0.38 ± 0.22 | 0.22 ± 0.21 | 0.36 ± 0.15 | | | | 0.04 ± 0.03 | 0.06 ± 0.04 | 0.06 ± 0.04 | 0.16 ± 0.16 |
| EV/BV _day0-15_ (mm³/mm³) | 0.0002 ± 0.0002 | 0.0003 ± 0.0003 | 0 ± 0 | 0.0001 ± 0.0002 | | | | 0.0002 ± 0.0002 | 0.0024 ± 0.0057 | 0.0003 ± 0.0003 | 0.0018 ± 0.0033 |
| ES/BS _day0-15_ (mm²/mm²) | 0.002 ± 0.002 | 0.003 ± 0.003 | 0 ± 0 | 0.002 ± 0.003 | | | | 0.002 ± 0.002 | 0.028 ± 0.064 | 0.006 ± 0.006 | 0.036 ± 0.066 |

**Supplementary Table 4:** Endocortical (Ec) and periosteal (Ps) bone parameters of the tibial midshaft determined by dynamic histomorphometry in 10- and 26-week-old LC and Sost KO male mice, exposed to axial tibial compression (left tibia loaded, right tibia nonloaded control). Data are given as mean ± SD. ANOVA: indicates an effect of (**a**) genotype, (**b**) age, (**c**) loading, (**d**) genotype & age, (**e**) genotype & loading, (**f**) age & loading, p < 0.05.

| **Parameters** | **10-week-old** | | | | **26-week-old** | | | |
| --- | --- | --- | --- | --- | --- | --- | --- | --- |
|  | **LC** | | ***Sost* KO** | | **LC** | | ***Sost* KO** | |
|  | **Control** (n=6) | **Loaded** (n=6) | **Control** (n=7) | **Loaded** (n=7) | **Control**  (n=7) | **Loaded** (n=7) | **Control** (n=6) | **Loaded** (n=6) |
| **Endocortical** |  |  |  |  |  |  |  |  |
| Ec.sLS/BS (%) a | 37.6±15.4 | 28.1±15.4 | 53.9±22.2 | 59.4±25.8 | 22.9±19.0 | 21.6±16.2 | 41.4±20.7 | 41.8±12.2 |
| Ec.dLS/BS (%) b | 32.9±18.2 | 40.3±18.2 | 42.3±25.5 | 37.1±14.6 | 11.5±5.9 | 8.8±6.5 | 14.2±7.5 | 22.1±11.5 |
| Ec.MS/BS (%) a, b | 51.6±21.6 | 54.3±21.6 | 69.2±14.7 | 62.7±8.6 | 22.9±14.5 | 19.6±12.4 | 34.9±14.5 | 43.0±11.8 |
| Ec.MAR (μm/day) b, d | 1.37±0.24 | 1.46±0.32 | 1.10±0.32 | 1.20±0.33 | 0.76±0.19 | 0.75±0.14 | 0.91±0.36 | 0.94±0.22 |
| Ec.BFR/BS (μm³/μm²/day) b | 0.74±0.33 | 0.82±0.41 | 0.76±0.26 | 0.77±0.27 | 0.17±0.11 | 0.14±0.08 | 0.29±0.09 | 0.41±0.16 |
| **Periosteal** |  |  |  |  |  |  |  |  |
| Ps.sLS/BS (%) b | 24.2±10.9 | 34.2±5.3 | 34.5±11.8 | 39.6±8.5 | 55.2±20.7 | 55.0±15.7 | 57.4±21.6 | 51.0±14.0 |
| Ps.dLS/BS (%) b | 38.6±26.1 | 43.3±22.0 | 36.7±25.3 | 29.7±19.9 | 19.0±9.2 | 24.6±19.8 | 13.8±4.4 | 21.3±14.0 |
| Ps.MS/BS (%) | 50.7±25.8 | 60.4±21.8 | 54.0±21.0 | 49.5±17.9 | 46.6±18.1 | 52.1±14.3 | 34.0±20.7 | 48.6±12.2 |
| Ps.MAR (μm/day) b | 1.66±0.99 | 1.60±0.86 | 0.94±0.26 | 1.20±0.35 | 0.67±0.14 | 0.72±0.21 | 0.89±0.18 | 0.91±0.31 |
| Ps.BFR/BS (μm³/μm²/day) | 1.04±1.01 | 1.09±0.83 | 0.54±0.33 | 0.62±0.37 | 0.31±0.13 | 0.38±0.17 | 0.39±0.16 | 0.47±0.29 |
